# Microbial nitrogen bubble formation in porous media

**DOI:** 10.1016/j.heliyon.2024.e32671

**Published:** 2024-06-07

**Authors:** Daehyun Kim, Hojeong Kang, Leon A. van Paassen, Liya Wang, Tae Sup Yun, Toshiro Hata

**Affiliations:** a School of Civil and Environmental Engineering, Yonsei University, Seoul, Republic of Korea; b Department of Civil and Environmental Engineering, Hiroshima University, Hiroshima, Japan; c School of Sustainable Engineering and the Built Environment, Arizona State University, Tempe, AZ, USA; d Key Laboratory of Roads and Railway Engineering Safety Control, Shijiazhuang Tiedao University, Shijiazhuang, China

**Keywords:** Nitrogen gas, Bubble formation, Denitrification, Biogeochemical process, Monitoring and characterization, Porous media

## Abstract

Microbially induced nitrogen (N_2_) gas bubbles can desaturate subsurface areas and thus have been considered as an alternative ground improvement technique for mitigating soil liquefaction potential caused by earthquakes. However, the detailed mechanisms of subsurface N_2_ bubbles are not well understood and remain a subject of ongoing research. In this study, a transparent microfluidic device was utilized to mimic biological N_2_ gas bubble formation by nitrate-reducing bacteria and to visually characterize the entire process. During N_2_ gas formation, a limited number of bubble nucleation sites were identified, which gradually expanded upward through the preferential pore channels. N_2_ gas bubbles tended to create interconnected gas pockets rather than existing as evenly distributed small gas cavities. The degree of water saturation gradually reduced over a week as the bubbles were produced. The gas ganglia repeatedly grew until they reached the top boundary, which triggered a drastic expulsion of bubbles by ebullition. Despite fluctuations in saturation level, the residual saturation was maintained at around 73 %. Comparative experimental case studies of CO_2_ gas bubble formation were conducted to identify contrasting gas formation mechanisms. CO_2_ gas bubbles were generated via the abiotic decompression of a supersaturated CO_2_ solution under two distinct rates of pressure reduction. Rapid CO_2_ bubble formation led to uniform nucleation and 41 % residual saturation, while slower formation yielded 35 % due to stable liquid displacement by the gas front. This study highlights the potential of the microfluidic device as an experimental tool for visualizing subsurface gas formation mechanisms. The insights gained could further enhance and optimize geotechnical applications involving gas formation in highly saturated soils.

## Highlights

• Microfluidic tests reveal subsurface microbial N_2_ bubble formation mechanisms.
• Bubble formation mechanisms and saturation levels vary with gas type and formation rate.
• Biogenic N_2_ bubble formation favors bubble growth; rapid CO_2_ disperses more evenly.
• The implications and necessary cautions when using microfluidic devices are discussed.

## 1 Introduction

Subsurface denitrification is a part of the nitrogen (N_2_) cycle where bacteria convert nitrate into nitrogen gas, thus preventing excess nitrogen from polluting water and atmospheric systems. Microbial nitrogen gas bubbles can also desaturate subsurface areas and have been considered as an innovative ground improvement technique for mitigating soil liquefaction potential caused by earthquakes [[1], [2], [3]].

Liquefaction occurs when the shear strength of soil decreases dramatically due to increased pore pressure in saturated and/or cohesionless soils under cyclic loading conditions, leading to soil failure and significant infrastructure damage. Desaturating soil can significantly enhance liquefaction resistance, prompting an investigation into various desaturation techniques, such as injecting air [4], employing water electrolysis [5], using sand compaction piles [6], and applying sodium perborate [7]. Even a minor reduction in soil water saturation (for instance, 1–10 %) can substantially improve the resistance of soil to cyclic loading conditions [[8], [9], [10], [11]]. For example, research by Okamura and Soga [9] showed that lowering the water saturation in the sand to 90 % nearly doubled its cyclic resistance, while a further decrease to 70 % yielded only a slight additional improvement [3].

Microbial denitrification, which reduces nitrate (${\text{NO}}_{3}^{‐}$) to nitrogen gas (N_2_), is being explored as an efficient desaturation technique. It involves the oxidation of organic matter or carbon sources where organic carbon compounds are broken down, and electrons are transferred to nitrate, facilitating its reduction to nitrogen [1,2,12]. The breakdown of organic carbon not only provides the necessary energy for the denitrification process but also leads to the formation of carbon dioxide (CO_2_) as a byproduct. The resulting nitrogen and carbon dioxide gases get trapped in the soil and cause desaturation. The chemically inert and minimally water-soluble N_2_ gas, with a solubility of 0.017 g/L at 25 °C under atmospheric pressure, is advantageous for maintaining soil desaturation as it can remain stable and persist for extended periods. Previous experimental studies have shown that N_2_ gas formation via microbial denitrification in soil columns began within a week [3,12,13] and persisted for over a year (more than 400 days), during which the water saturation level could be reduced to 80–95 % [3,14,15]. In these studies, saturation was experimentally measured by tracking the reduction in substrate concentration or by observing changes in soil volume and the amount of liquid displaced by gas production.

Several efforts have been made to estimate the reduction in water saturation yielded by microbial N_2_ gas formation. These efforts involve theoretical calculations of the volume of N_2_ gas produced at varying depths and pressures, based on principles from Henry's law and the ideal gas law [16,17]. However, both numerical and experimental approaches typically presume a consistent spread of gas bubbles throughout the soil matrix and overlook the nucleation and growth kinetics of gas bubbles. He et al. [18] mentioned that denitrifying bacteria might produce small, inert N_2_ gas bubbles that tend to be uniformly distributed. This uniform distribution is likely because bacterial cells and substrates can easily disperse while the low-viscosity reactive solution is being flushed.

Previous studies have examined the patterns of N_2_ gas bubble formation through microbial denitrification using visual observation methods. He [14] noted that substantial gas pockets formed through microbial denitrification which were unevenly spread as shown by X-ray Computed Tomography (XCT) sectional images. Pham et al. [13] also used XCT imaging to examine microbial N_2_ gas distribution at the column scale. They observed that the gas bubbles predominantly concentrated in the coarse cap layers, whereas some gas formed cracks in the column center consisting of finer grains. While it is widely recognized that even a minimal amount of gas within the soil pores can markedly alter soil properties, the scattering patterns and the durability of the produced microbial N_2_ gas bubbles remain ambiguous.

This study aims to investigate the intricate processes involved in microbial N_2_ gas bubble formation within porous media, with a specific focus on the interplay of nucleation, growth, movement, and displacement patterns, as well as the resultant gas distribution and saturation level. This process may present variations from the classical bubble nucleation and growth theories. To gain a clearer understanding of these mechanisms, a series of experiments using a transparent microfluidic device were carried out. These experiments visually characterize a distinct mechanism of microbial N_2_ gas bubble formation, contrasting it with CO_2_ gas bubble formations under varying gas formation rates and pressure conditions.

## 2 Background on bubble formation in porous media

Research into the formation and release of bubbles typically emphasizes fundamental processes including bubble formation, movement, and expansion. These processes are influenced by various factors such as the characteristics of gas and liquid, the required energy for phase transformation, the solubility of gas in liquid, and the geometric properties of the pore structure [[19], [20], [21], [22]].

When gas concentrations in a solution surpass a critical threshold far above the stable equilibrium concentration determined by Henry's law [23], gas bubbles begin to form:

(Eq. 1) $$C^{*}=\frac{P}{K_{H}}$$

Here, C* indicates the stable concentration of gas dissolved in the liquid, P is the partial pressure of the gas, and K_H_ is Henry's constant. The propensity for bubble formation is greater in the presence of irregular or textured surfaces, which serve as catalytic sites for nucleation, compared to a perfectly homogeneous liquid environment [[23], [24], [25]]. The stability of these nascent bubbles is governed by a critical size, the determination of which is influenced by the Young-Laplace equation [26]:

(Eq. 2) $$\Delta P=P_{g}‐P_{w}=\frac{2\gamma }{R}$$

In this expression, ΔP denotes the pressure difference across the bubble boundary, P_g_ and P_w_ are the pressures within the gas and the surrounding water respectively, γ represents the interfacial tension, and R represents the bubble's radius.

Following nucleation, bubbles grow by either absorbing more gas due to pressure reductions or through direct gas transfer, as described by Boyle's Law [27] stating that the volume, V, varies proportionally to the inverse of the pressure, P:

(Eq. 3) $$V\propto \frac{1}{P}$$

Reductions in pressure not only allow existing bubbles to expand but also lower gas solubility, potentially increasing gas exchange driven by a concentration gradient:

(Eq. 4) $$J=k_{L}A\left(C_{L}‐C^{*}\right)$$

Here, J is the gas transfer rate, k_L_ represents the mass transfer constant, A denotes the boundary surface, and C_L_ indicates the concentration in the aqueous phase.

To understand gas bubble behavior in porous environments, models such as those by Mahabadi et al. [22,27] simulate micro-bubbles starting as random distributions. These bubbles rise through the porous matrix due to buoyancy, growing by absorbing gas or merging with other bubbles. The migration of these bubbles is often unrestricted when they are smaller than the pore throats but becomes inhibited once they exceed this size, potentially leading to entrapment [28].

Increased gas bubble volumes can cause entrapment in pores, yet trapped bubbles can still displace liquid. The dynamic interaction between gas and liquid phases, important in fields including oil and gas extraction, carbon dioxide storage, and environmental remediation, follows known displacement patterns-stable displacement, viscous fingering, and capillary fingering, affected by fluid viscosity ratios, interfacial tensions, and flow dynamics [[29], [30], [31], [32], [33]].

Key dimensionless numbers used to analyze these interactions include the Capillary number, Ca, which balances viscous forces against surface tension [32]:

(Eq. 5) $$\text{Ca}=\frac{\nu \mu_{\text{inv}}}{\gamma }$$

and the viscosity ratio, M, indicative of displacement characteristics [32]:

(Eq. 6) $$M=\frac{\mu_{\text{inv}}}{\mu_{\mathrm{def}}}$$

where ν is flow rate [m^3^⋅s^−1^] and μ_inv_ and μ_def_ represent the viscosities of the invading and defending fluids [Pa·s], correspondingly. The Bond number [34], Bo, contrasts gravitational forces with capillary forces, shaping the bubbles' movement and morphology in the pore space:

(Eq. 7) $$\text{Bo}=\frac{\Delta \rho gL^{2}}{\gamma }$$

In this formula, Δρ quantifies the density differential between the gas and liquid phases [kg⋅m^-3^]. The variable g denotes the acceleration of gravity [m⋅s^-2^], and L specifies the characteristic length in meters, which typically corresponds to the curvature radius at the interface between gas and water. A lower Bond number suggests the dominance of capillary forces, preserving spherical bubble shapes, whereas higher values facilitate bubble deformation [32]. The Bond number additionally influences the behavior of trapped bubbles within pores. When these bubbles completely occupy a pore, they demonstrate a ‘slug’ type movement while navigating through narrow pore throats. Such slug movements do not occur within capillary tubes filled with water when the Bond number is below 3.37, highlighting the role of capillary forces in restricting bubble mobility under certain conditions [35,36].

## 3 Material and methods

### 3.1 Microfluidic channel

**Fig. 1:**
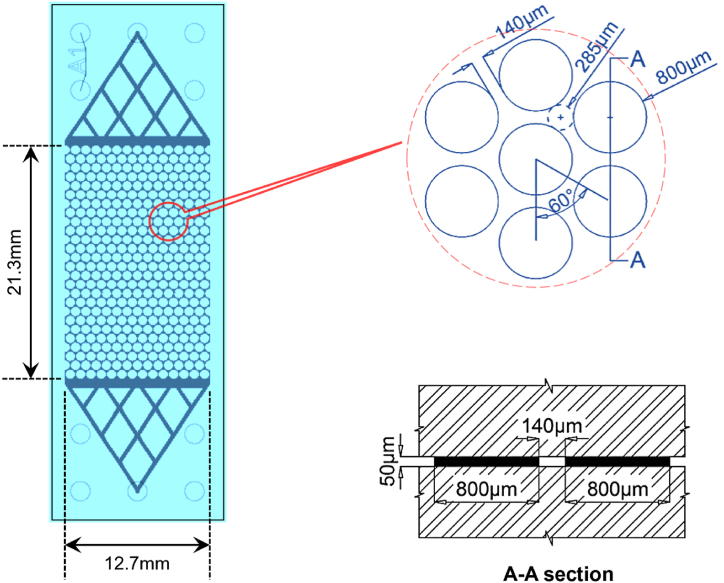
Geometry of a homogeneously patterned microfluidic channel.

A transparent two-dimensional microfluidic channel, produced by Micronit B·V., was engineered to replicate the packing of uniformly sized circular particles. The channel measures 21.3 mm by 12.7 mm with a pore depth of 50 μm. It includes 377 uniformly sized circular grains, each 800 μm in diameter, spaced with pore throats of 140 μm between them (Fig. 1).

### 3.2 Microbial N_2_ gas bubble formation

**Fig. 2:**
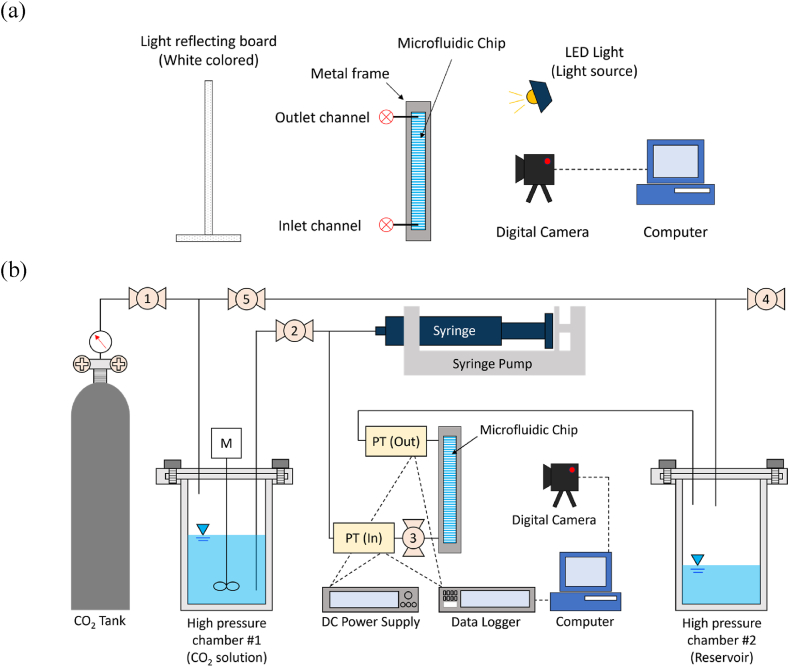
Experimental configurations for gas bubble forming tests in the microfluidic chip: (a) microbial N_2_ gas, and (b) CO_2_ gas.

For the study on microbial N_2_ gas generation in a microfluidic channel, a substrate solution was prepared and enriched with a diverse culture of nitrate-reducing bacteria. This culture was enhanced using garden soil collected from Arizona State University (Tempe campus) as the bacterial source. The solution composition included 12 mmol per liter of calcium acetate (Ca(C_2_H_3_O_2_)_2_) and 10 mmol per liter of calcium nitrate (Ca(NO_3_)_2_), with an addition of 0.5 mL/L of trace metal solution that contained 0.5 % weight/volume of CuSO_4_, FeCl_3_, MnCl_2_, and Na_2_MoO_4_·2H_2_O to foster bacterial growth, as referenced in prior research [3]. Fig. 2a visually details the setup used in this experiment. The microfluidic device was installed vertically within a metal frame holder to ensure stability. Approximately 5 mL of the formulated solution was injected through the lower inlet, effectively saturating the pore network of the microfluidic channel. To maintain the integrity of the experiment, the lower inlet was tightly sealed to eliminate any potential leaks, and the upper outlet was partially sealed to reduce the loss of solution through evaporation and to facilitate the expulsion of liquid by the generated gas bubbles. The experimental conditions were maintained for 28 days, in alignment with established protocols [[3], [37], [38]].

### 3.3 CO_2_ gas bubble formation via decompression of the supersaturated CO_2_ solution

A system capable of withstanding high pressures was built specifically for conducting CO_2_ gas bubble formation tests within a microfluidic channel, as depicted in Fig. 2b. This setup included a CO_2_ reservoir, a high-pressure resistant chamber (referred to as Chamber #1) for the supersaturation of CO_2_ in water, and a syringe coupled with a pump to transfer the supersaturated CO_2_ solution from Chamber #1 into the microfluidic channel. Additionally, a secondary high-pressure resistant chamber (Chamber #2) was used to collect the effluent from the microfluidic channel outlet. The system was segmented using five strategically placed valves and was completely sealed from external atmospheric influence by closing Valve #4. To accurately track changes in pressure throughout the experiment, two pressure transducers were installed at both the inlet and outlet of the microfluidic channel, powered by a 28 V DC power supply (E3645A, Agilent). Data regarding pressure fluctuations were recorded at 5-s intervals using a data logger (34972A, Keysight) connected to a computer. The experiment procedure was as follows:

*Preparation of the supersaturated CO*_*2*_*solution.* The process began by closing Valves #3 and #4 and opening the CO_2_ gas reservoir to set the pressure at 1 MPa, facilitating the dissolution of CO_2_ into deionized (DI) water within Chamber #1. This pressure was maintained for a day (24 h), enabling CO_2_ to fully saturate the DI water. Following this, Valve #1 was shut to segregate the CO_2_ tank from the system, securing the supersaturated solution in Chamber #1.

*Extraction of the supersaturated CO*_*2*_*solution.* The supersaturated solution was then extracted using a syringe once Valve #5 was closed. 5 mL of this solution was injected into the channel via the lower inlet after the sealing of Valve #2 and the activation of Valve #3. To guarantee complete saturation, the amount of the introduced solution exceeded the total capacity of the channel pores and its associated tubing, forcing any surplus liquid out through the upper exit into Chamber #2.

*Decompression of the system.* The decompression of the system started with the closure of Valve #3 and the activation of Valve #4 to begin depressurizing the microfluidic channel. The depressurization rates were manually regulated by adjusting the rotational angle of Valve #4, with rates precisely calculated based on the initial pressure gradient recorded by the outlet PT sensor. The rates were set at −25 kPa/s for rapid decompression and −1 kPa/s for slower decompression.

### 3.4 Monitoring of gas formation

To monitor gas formation, time interval photography was utilized, employing a Nikon D5200 digital camera fitted with a microlens (AF-S Micro NIKKOR 60 mm f/2.8G ED, Nikon). The setup included four LED lights (JANSJÖ, IKEA), accompanied by a panel designed to enhance the lighting conditions. In the experiments focused on microbial N_2_ gas generation, snapshots were taken at 10-minute intervals with a clarity of 1920 × 1080 pixels, allowing for the detailed observation of gas dynamics in the microfluidic channel. Meanwhile, the CO_2_ gas formation was captured using video recording with a frame rate set at 25 frames per second, consistently using a resolution of 1920 × 1080 pixels.

### 3.5 Algorithm for phase segmentation from the experimental image

**Fig. 3:**
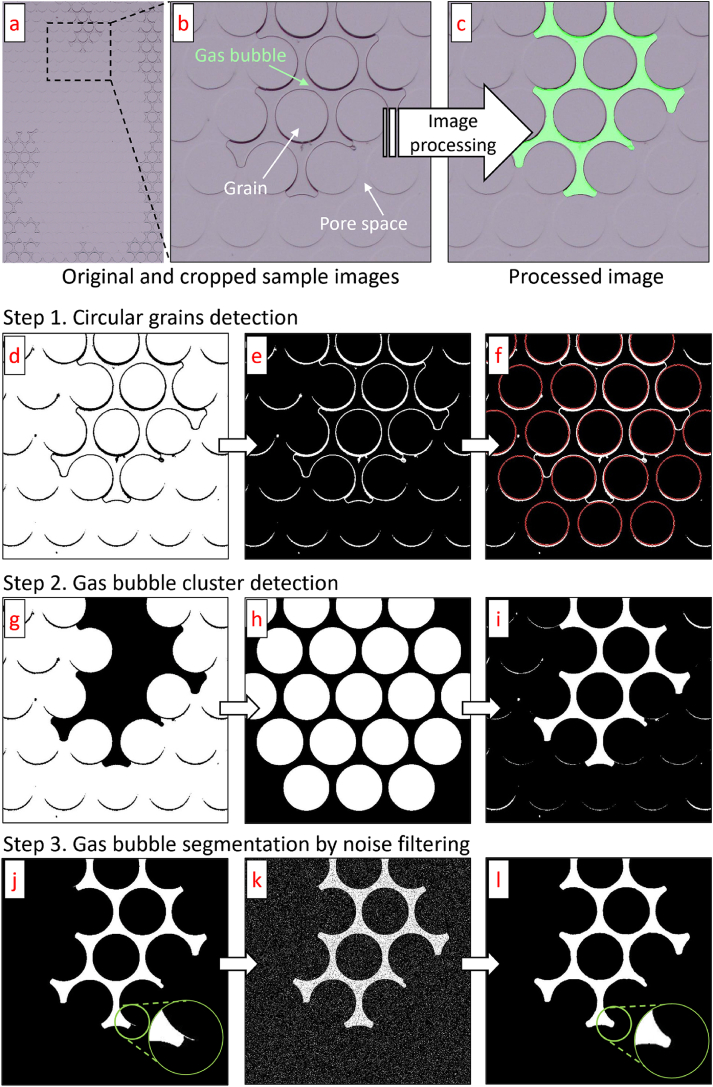
Gas bubble segmentation procedure: (a) sample raw image (after 244 h), (b) cropped zone from the sample image, (c) processed result image, (d) binarization via Otsu's method, (e) inverted image, (f) detection of circular grains, (g) bubble cluster detection, (h) circular grain mask map, (i) roughly segmented bubble cluster, (j) removal of small noise by size thresholding, (k) addition of artificial noise for noise filtering, and (l) output after applying a 2D median filter.

Fig. 3a showcases an example of a captured image from the microbial N_2_ gas bubble formation experiment after 244 hours (about 10 days). The raw image features various phases including grains, gas bubbles, and a liquid solution filling the pore space. These elements are more clearly detailed in a clipped section of the raw image (Fig. 3b). To identify gas bubbles and calculate their volume for saturation analysis, an advanced image processing algorithm was developed using MATLAB (R2023a). Fig. 3c illustrates an example of the processed result, where gas pockets are accurately marked in light green. This algorithm involved several steps, starting with the conversion of color images to binary format, followed by segmenting the visual data into different phases: solids, liquid, and gas.

Initially, the full-color raw image, composed of three layers of matrices (Red, Green, and Blue), was converted to grayscale. This grayscale image, now an 8-bit intensity image, simplifies the data into a single matrix which is easier to analyze. The grayscale image was then converted to a binary format (black and white) through the application of Otsu's method [39], executed with ‘imbinarize’ command (Fig. 3d). Adaptive thresholding was applied to adjust brightness variations due to uneven illumination.

The binary image not only highlighted the gas bubbles but also the edges of circular grains. To isolate the grains, the image was inverted (Fig. 3e), and ‘imfindcircle’ command in MATLAB was used to pinpoint these grains, marked by red lines in Fig. 3f. The identified grains were then used to create a separate binary image showing only the grains.

The binary image showed large clusters of gas bubbles with well-defined edges (Fig. 3d). Using the ‘bwareaopen’ command in MATLAB, smaller connected objects were removed, leaving only significant lumps including a bubble cluster and circular grains (Fig. 3g). These detected lumps were then masked by the circular grains (Fig. 3h), resulting in the separation of the gas bubble cluster (Fig. 3i).

The small noises after the initial detection steps were addressed by setting a size threshold (Fig. 3j). The remaining minor artifacts were tackled by adding artificial noise (Fig. 3k) and applying a 2D median filter to clean up the final image, which displayed the bubble clusters distinctly (Fig. 3l).

The total volume of gas bubbles was estimated by counting the pixels within these clusters, assuming full occupation of the microfluidic channel's depth. This automated image processing approach offered substantial benefits over manual methods by ensuring consistent segmentation and reducing the time required to analyze numerous images. Modifications to the algorithm parameters were occasionally necessary to accommodate variations in image color and bubble characteristics.

## 4 Results and discussion

### 4.1 Microbial N_2_ gas formation via denitrification

**Fig. 4:**
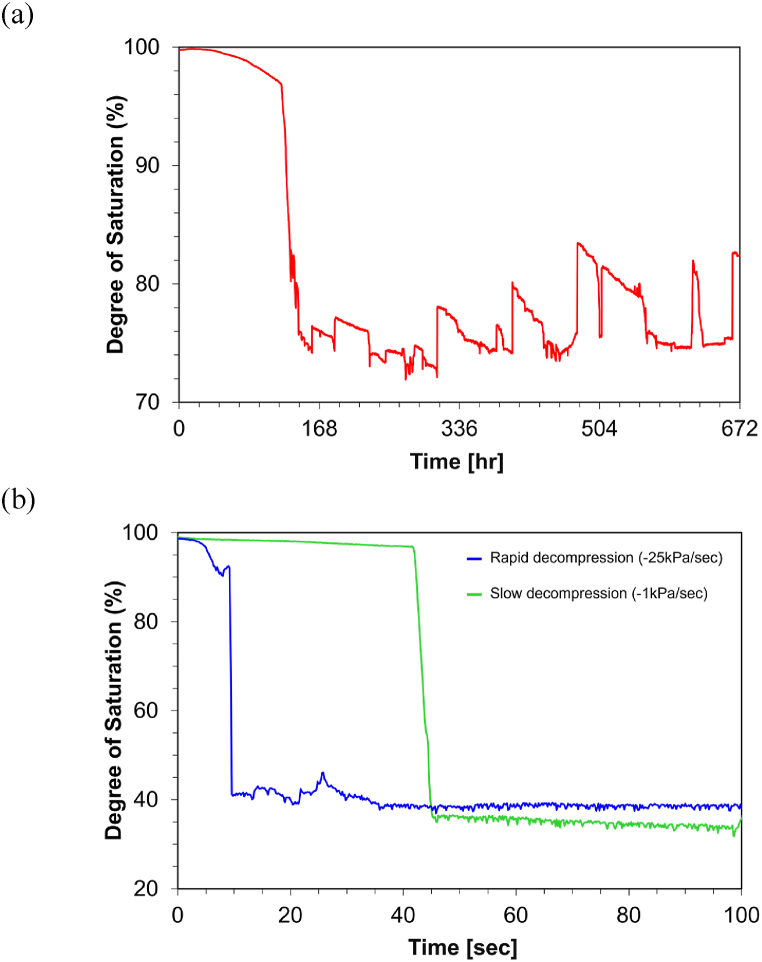
The degree of saturation changes within the microfluidic chip: (a) microbial N_2_ gas bubble formation and (b) CO_2_ gas bubble formation under decompression.

**Fig. 5:**
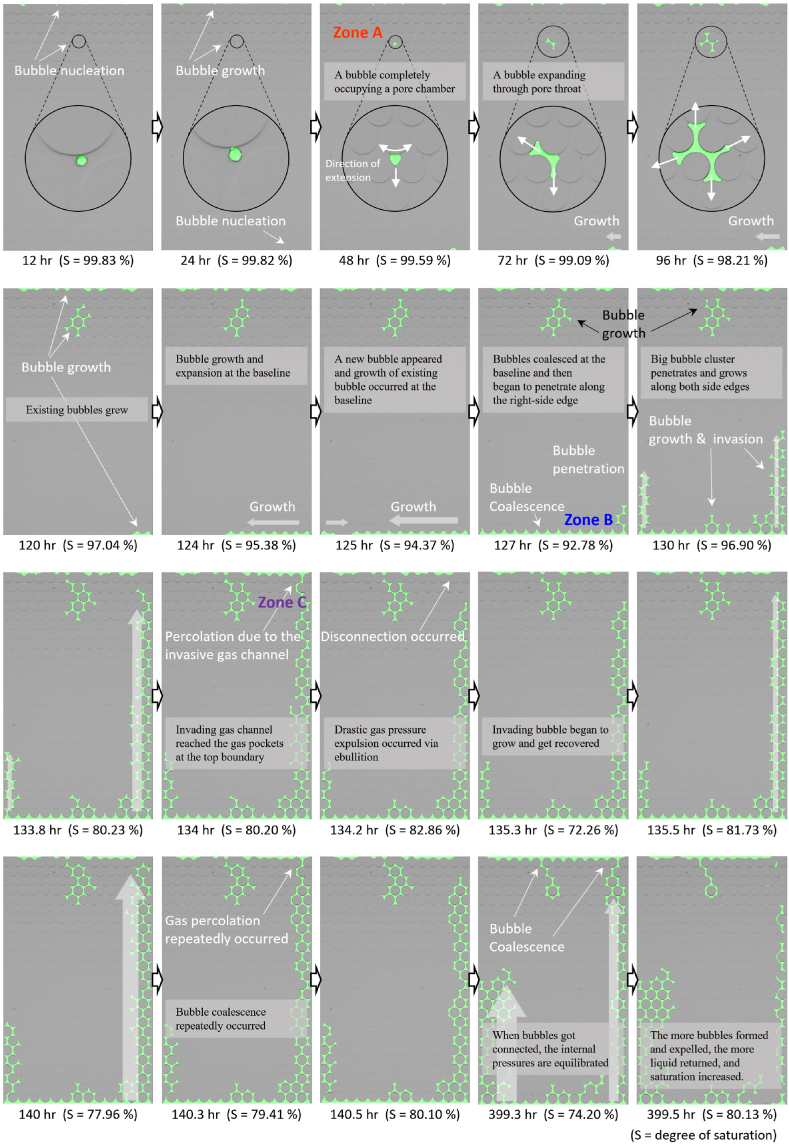
Microbial N_2_ gas bubble formation and growth mechanisms within the microfluidic chip.

Fig. 4a shows the changes in the degrees of saturation over time during denitrification within the microfluidic chip. Fig. 5 presents selected images illustrating various processes of N_2_ gas bubble development.

**Fig. 6:**
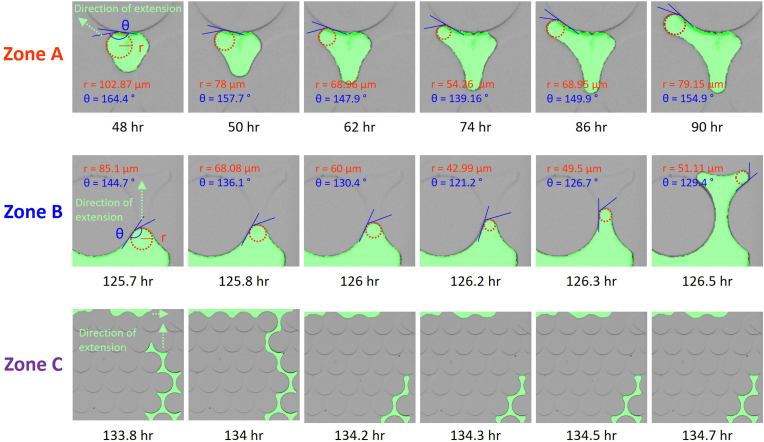
Microbial N_2_ gas bubble formation and growth mechanisms at the highlighted zone.

After the first 24 hours, the initial bubble emerged at the top part of the channel, highlighted within a black circle (Figs. 5, 12 hr). It originated at the edge of a circular grain, conforming to predictions regarding the energy barrier for heterogeneous nucleation within the bulk liquid [40,41]. The nucleated bubble exhibited a perfectly round in a 2D frontal view and had a radius of 50.8 μm. It expanded symmetrically as the microbial denitrification progressed, maintaining its form up to the point where it occupied the largest available area between a trio of adjacent particles (Figs. 5, 48 hr). As it approached adjacent pore throats under increasing gas pressure, the bubble started penetrating through these narrow passages, a segment marked as ‘Zone A’ in Fig. 5. This movement is further detailed in Fig. 6a with an enlarged view showing that the bubble's radius reduced from 102.87 μm to 54.26 μm to pass through the pore throats. Based on the Young-Laplace formula (Eq. 1), the capillary pressure, reciprocal to the radius of the bubble, decreases as the bubble squeezes through the tightest section of the pore throat and rapidly fills the nearby pore space, a phenomenon known as Haines Jumps [42,43].

In this study, bubble expansion within the microfluidic channel occurred gradually due to the large size of the pore throats (140 μm) relative to the channel thickness (50 μm). Owing to this design feature, capillary pressure remained controlled by the narrowest radius within the system and was consistent. The expansion behavior and movement direction of the gas bubbles were primarily influenced by the pore throat size and buoyant forces. Notably, a lower ratio of pore chamber to pore throat size facilitated a gradual and uniform isotropic expansion, while buoyancy aided the upward growth and migration of the bubbles.

Meanwhile, gas bubbles emerged within the outlet (upper) and inlet (lower) channels, connected to the lattice-shaped large pore spaces designed to ensure uniform distribution of the injected fluid as shown in Fig. 1a. These bubbles extended sideways and ultimately coalesced into a substantial gas pocket that entirely occupied the inlet channel (Fig. 5, 72–127 hr). Once the gas pocket filled the inlet, it rapidly ascended along the channel's side walls as detailed in Zone B of Fig. 5, Fig. 6. During this upward movement, similar to the initial bubble expansion noted in Zone A, the radius of curvature at the constricted region decreased from 85.1 μm to 42.99 μm.

The pattern of residual gas distribution observed in this experiment corresponds to results from prior studies that examined microbial N_2_ gas formation through denitrification in soil columns [13,14]. These studies indicated that gas bubbles primarily accumulated in large pockets within coarser grain layers. Once the channels along the lateral pores of the microfluidic device were filled with gas, rapid venting occurred at the upper ends, temporarily leaving isolated gas pockets along the sides. These pockets gradually merged again as more gas was produced, with the cycle of quick release followed by slow migration and reconnection repeating throughout the experiment. This recurring pattern is referred to as ebullition, as depicted in Zone C of Fig. 5, Fig. 6.

Throughout the test, as the gas bubbles expanded, the saturation level within the microfluidic channel decreased. Initially, saturation was complete at 100 %, as the channel was entirely filled with the substrate mixture. Fig. 4a illustrates the changes in saturation over time. The steady reduction in the degree of saturation over the initial 125 hours was primarily due to the expansion of the central bubble. The rate of bubble growth accelerated, in line with biological activities that increase biomass and enhance biogas production. Following 125 hours, a sharp decline in saturation was observed dropping to a minimum of 75 % due to the swift expansion and invasion of a gas bubble through the lower inlet and side edges (Fig. 4, Fig. 5). This rapid progression was also accelerated by biological activities in the lattice pores at the inlet of the microfluidic device, which formed gas bubbles and expelled liquid or gas upward.

The degree of saturation began to show pronounced fluctuations. As the invading gas reached the upper gas pocket, saturation levels spiked with each venting episode, then fell as new gas accumulated and ascended. These fluctuations, ranging from 72 % to 85 %, repeated several times and were highlighted in Fig. 4a. Each peak, reaching up to 84.4 %, corresponded with an increase in gas volume being vented upwards and the returning liquid volume as isolated gas pockets reconnected. This phenomenon was also attributed to the liquid originally present in the upper lattice pores and the connected tubing at the onset of the experiment. As more gas bubbles formed through the denitrification process, more liquid was expelled out of the monitored boundary and recollected as part of the ebullition phenomena. The observed saturation changes closely align with those documented in previous research emphasizing the cyclical nature of gas and liquid movements within the soil system [13].

### 4.2 CO_2_ gas formation via rapid and slow decompression

**Fig. 7:**
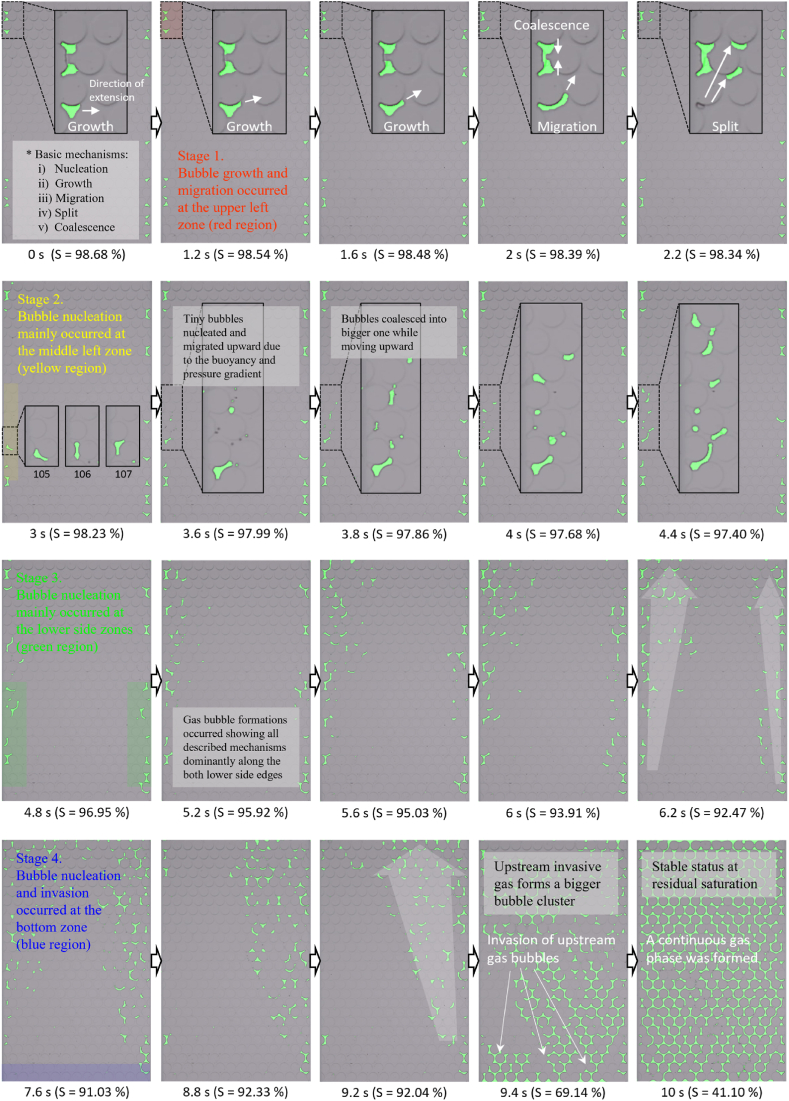
CO_2_ gas bubble formation and growth mechanisms under rapid decompression within the microfluidic chip.

The experiment on CO_2_ gas bubble formation using rapid decompression revealed distinct bubble formation and migration behaviors compared to those observed in the microbial N_2_ gas bubble formation test. These differences notably influenced the residual saturation levels and the patterns of gas distribution within the microfluidic channel. Initially, during the solution injection, some air bubbles became entrapped along both sides of the channels, impacting the early stages of CO_2_ gas phase changes (as seen in Step 1 of Fig. 7).

Fig. 7 illustrates that rapid decompression initiated the nucleation, growth, and migration of numerous small CO_2_ bubbles across the channel. In the first few seconds, the formation and expansion of these bubbles were primarily concentrated along the channel sides (Step 2 in Fig. 7). The growth of these bubbles was driven by pressure drop as per the ideal gas law [44]. The bubbles generally ascended vertically consistent with earlier research on bubble behavior [12,22]. The driving forces of this upward migration included buoyancy, pressure differentials, and the liquid flow from the bottom to the top of the channel.

Splitting and coalescence of bubbles were observed, similar to the finding of Mahabadi and van Paassen [45] as particularly indicated in the black-boxed area of Fig. 7. As the pressure continued to decrease, additional bubbles nucleated, especially in the upper sections of the channel (Fig. 7). In the initial 4.8 s, the saturation level slowly declined to reach 96.95 % due to the continuous nucleation and growth of bubbles (blue line in Fig. 4b). Between 5.2 and 5.6 s, the saturation level stabilized (95.92–95.03 %) as the quantity of newly formed bubbles matched the amount of gas bubbles exiting throughout the channel (Step 3 in Fig. 7). Between 7.6 and 9.6 s, a sharp decline in saturation was observed as bubbles rapidly migrated upward through several preferential pore channels (noted at 9.4 s in Fig. 7), dropping to 41.2 % (Step 4 in Fig. 7). Once upstream invasive bubbles reached the top outlet, it formed a continuous phase from the bottom to the top of the channel (noted at 10 s in Fig. 7). Although some minor nucleation and migration of bubble occurred, the degree of saturation remained constant after 9.6 s, stabilized at approximately 41 % for the remainder of the experiment.

**Fig. 8:**
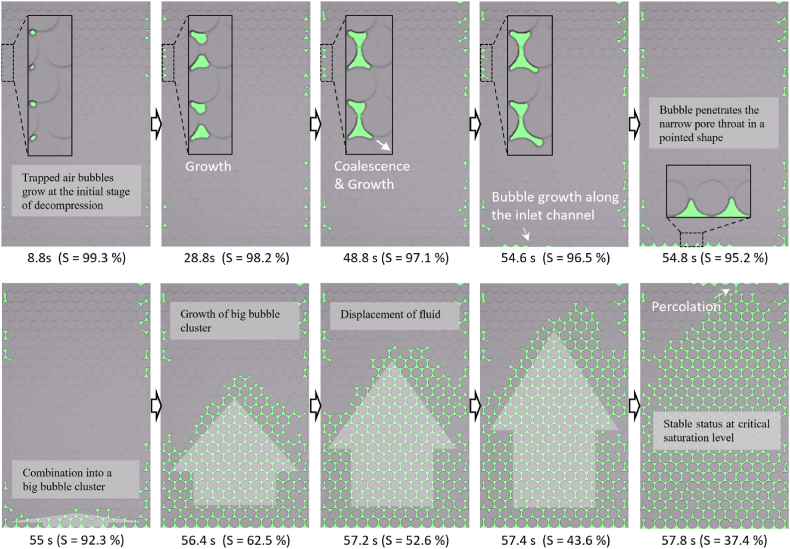
CO_2_ gas bubble formation and growth mechanisms under slower decompression within the microfluidic chip.

In contrast, the slow decompression case exhibited different CO_2_ gas bubble formation patterns, primarily characterized by gas bubble displacement as seen in Fig. 8. Similar to the rapid decompression scenario, a few bubbles trapped during the initial injection served as nucleation points once decompression commenced. Gas bubbles were observed forming at the bottom after 54 s, and a stable gas front progressively ascended, displacing the liquid until it filled the channel from bottom to top by 57.8 s. This resulted in an unbroken phase of gas bubble throughout the channel and a residual saturation level of around 35 %.

### 4.3 Comparative interpretation and constraints

A comparison review of the three experimental cases highlights significant variances in gas bubble formation and migration processes:

1. Microbial N_2_ gas bubble formation exhibited gradual growth of nucleated bubbles, which led to a non-uniform gas distribution with a residual saturation between 72 % and 85 %.
2. Rapid decompression induced quick nucleation and growth of numerous bubbles, with their upward movement via multiple pathways leading to an evenly spread gas phase and a residual saturation of approximately 41 %.
3. Slow decompression showed no initial nucleation of gas bubbles. Instead, restrained growth, followed by steady displacement resulted in a continuous gas phase, with a residual saturation of about 35 %.

Gas bubble displacement emerged as the primary mechanism affecting saturation reduction across all scenarios. The displacement mechanisms within porous environments are affected by factors such as the Capillary number, viscosity ratios, and Bond number. The assumptions include water having a viscosity of 8.9 × 10^−4^ Pa⋅s [46], nitrogen and carbon dioxide gases having viscosities of 1.76 × 10^−5^ Pa⋅s [47] and 1.74 × 10^−5^ Pa⋅s [48] respectively, resulting in a viscosity ratio of 0.0157. Calculated gas flow rates stand at 9.3 × 10^−11^, 2.5 × 10^−7^, and 1.4 × 10^−4^ m^3^⋅s^−1^ for each respective case. These rates are determined by calculating the change in volume of the gas moving through the channel's cross section and the cumulative duration of displacement. Capillary number values are derived at 2.30 × 10^−12^, 6.20 × 10^−9^, and 3.41 × 10^−6^ for each experiment based on an assumed interfacial tension of 72 N⋅m^−1^. Such low values indicate that capillary or viscous fingering might dominate as the primary displacement mechanism [49,50].

Considering a characteristic length (a variable, L, in Eq. (7)) of 2.5 × 10^−5^ m, which aligns with the minimum bubble diameter constrained by the channel thickness, and accounting for a gas density of 0.8 kg⋅m^−3^ along with a density of water at 1 × 10^3^ kg⋅m^−3^, the Bond number is calculated to be 8.5 × 10^−5^. This value is below 3.37, which indicates that trapped bubbles are unlikely to move solely due to buoyancy. Rather, the movement of larger gas bubbles is likely to occur through bubble growth or by the kinetics and hydraulic pressures of incoming fluids [35,36].

Despite identifying distinct gas bubble creating and movement processes, and their relationship with the rate of gas production, residual saturation levels, and gas distribution patterns, the detected mechanisms for bubble displacement do not fully correspond to theoretical anticipations. This discrepancy could arise from constraints within the experimental setup and procedures. For instance, the microfluidic channel's thickness being less than the diameter of the pore throat might interrupt gas bubbles ascent, leading to a continuous movement without Haines jumps. The highly uniform geometric condition of the channel could also impact the displacement mechanisms, as indicated by Mahabadi and van Paassen [45]. To closely simulate subsurface bubble formation mechanisms considering the pore size variability in actual soil, adjustments to the pore structure are advised.

Additional overlooked aspects are fine-grained soils and the potential layering deposits in nature. In such soils, physical constraints may inhibit the formation of microbial N_2_ gas bubbles by restricting microbial activities or migrations. Moreover, the tiny pores could destabilize bubbles, as higher gas pressure and the concentration at equilibrium are related to diminished bubble sizes. Conversely, produced gas bubbles could create macro-sized pores or lead to fractures [16]. Deposit layers could also obstruct gas from ascending, directing it instead to spread downward or laterally through coarser layers, potentially resulting in varying saturation levels depending on pore dimensions.

While a single experimental case of microbial N_2_ gas bubble formation was introduced in this research, subsurface N_2_ gas formation is governed by a multifaceted interaction among soil physicochemical properties, including texture, moisture content, organic matter, and pH [51,52]. This is alongside geotechnical properties such as soil structure and compaction [53,54], as well as environmental factors like temperature, oxygen availability, and seasonal variations [55,56]. These interactions not only influence the formation and behavior of N_2_ gas bubbles but also their ecological and geotechnical impacts. Thus, given the intricate nature of these interactions, further in-depth research is needed to elucidate how microbial N_2_ gas bubbles affect soil structure and function, and to understand their broader environmental implications.

## 5 Conclusions

This research utilizing a microfluidic channel experimentally demonstrated the distinct mechanisms of microbial N_2_ gas bubble formation by comparing it to CO_2_ gas bubble formations under different decompression conditions. In regular porous media, microbial N_2_ gas forms at a relatively slow pace. This slower pace constrains the nucleation of new gas bubbles, instead promoting the growth of pre-existing ones. As these bubbles expand, they occupy multiple pore chambers, consistently reducing the degree of saturation while also rising due to buoyancy. The primary mechanism driving this reduction in saturation is the displacement of liquid by the ascending gas bubbles from below, resulting in a residual saturation value ranging between 72 % and 85 %. It is noteworthy that these observations align with other experimental studies conducted on sand columns.

In the scenario of rapid gas formation, particularly as observed with the decompression of a supersaturated CO_2_ solution, the gas bubbles are uniformly distributed across the microfluidic channel, resulting in a residual saturation at approximately 41 %. With slower CO_2_ gas formation, stable displacement of liquid remains the primary mechanism influencing a lower saturation level, observed at around 35 %.

This study highlights the effectiveness of microfluidic devices as a robust tool for visualizing and understanding the intricate processes of subsurface gas bubble formation. However, while the observed mechanisms broadly align with theoretical expectations, discrepancies arise due to the heterogeneity of the system and the mismatches between the pore throats and the channel height. These variances suggest the need for further exploration into the physical and theoretical aspects that govern these processes. Despite not focusing primarily on engineering properties or environmental implications, the findings from this study offer perspectives that could enhance the development of geotechnical and environmental applications related to subsurface gas bubble formations.

## Data availability statement

Data supporting the findings of this research can be provided by the corresponding author upon a valid and justified request.

## CRediT authorship contribution statement

**Daehyun Kim:** Writing – review & editing, Writing – original draft, Visualization, Validation, Project administration, Methodology, Conceptualization. **Hojeong Kang:** Writing – review & editing, Resources. **Leon A.van Paassen:** Writing – review & editing, Writing – original draft, Validation, Supervision, Resources, Funding acquisition, Formal analysis, Conceptualization. **Liya Wang:** Writing – review & editing. **Tae Sup Yun:** Writing – review & editing, Validation, Resources. **Toshiro Hata:** Writing – review & editing, Resources.

## Declaration of competing interest

The authors declare that they have no known competing financial interests or personal relationships that could have appeared to influence the work reported in this paper.
